# Space–time analysis of gravitropism in etiolated Arabidopsis hypocotyls using bioluminescence imaging of the *IAA19* promoter fusion with a destabilized luciferase reporter

**DOI:** 10.1007/s10265-017-0932-6

**Published:** 2017-04-10

**Authors:** Kotaro T. Yamamoto, Masaaki K. Watahiki, Jun Matsuzaki, Soichirou Satoh, Hisayo Shimizu

**Affiliations:** 1 Division of Biological Sciences, Faculty of Science, Hokkaido University, Sapporo, 060-0810 Japan; 2 Biosystems Science Course, Graduate School of Life Science, Hokkaido University, Sapporo, 060-0810 Japan; 3 Present Address: Center for Sustainable Resource Science, RIKEN, Tsurumi-ku, Yokohama, 230-0045 Japan; 4 Present Address: Graduate School of Life and Environmental Sciences, Kyoto Prefecture University, Sakyo-ku, Kyoto, 606-8522 Japan

**Keywords:** Arabidopsis, Auxin, Aux/IAA, Bioluminescence imaging, Gravitropism, Hypocotyl

## Abstract

Imaging analysis was carried out during the gravitropic response of etiolated Arabidopsis hypocotyls, using an *IAA19* promoter fusion of destabilized luciferase as a probe. From the bright-field images we obtained the local deflection angle to the vertical, *A*, local curvature, *C*, and the partial derivative of *C* with respect to time, $\partial C/\partial t$. These were determined every 19.9 µm along the curvilinear length of the hypocotyl, at ~10 min intervals over a period of ~6 h after turning hypocotyls through 90° to the horizontal. Similarly from the luminescence images we measured the luminescence intensity of the convex and concave flanks of the hypocotyl as well as along the median of the hypocotyl, to determine differential expression of auxin-inducible *IAA19*. Comparison of these parameters as a function of time and curvilinear length shows that the gravitropic response is composed of three successive elements: the first and second curving responses and a decurving response (autostraightening). The maximum of the first curving response occurs when *A* is 76° along the entire length of the hypocotyl, suggesting that *A* is the sole determinant in this response; in contrast, the decurving response is a function of both *A* and *C*, as predicted by the newly-proposed graviproprioception model (Bastien et al., Proc Natl Acad Sci USA 110:755–760, 2013). Further, differential expression of *IAA19*, with higher expression in the convex flank, is observed at *A* = 44°, and follows the Sachs’ sine law. This also suggests that IAA19 is not involved in the first curving response. In summary, the gravitropic response of *Arabidopsis* hypocotyls consists of multiple elements that are each determined by separate principles.

**Electronic supplementary material:**

The online version of this article (doi:10.1007/s10265-017-0932-6) contains supplementary material, which is available to authorized users.

## Introduction

The gravitropic response is one of the most fundamental growth responses in plants. By orienting the plant towards the vertical, it helps to increase efficiency in photosynthesis and absorption of water and nutrients. Except for several kinematic studies (Bastien et al. 2013; Cosgrove 1990; Coutand et al. 2007; Meškauskas et al. 1999; Miller et al. 2007), the gravitropic response has usually been studied by measuring the change in the deflection angle of the organ tip. However, shoot gravitropism in particular should be examined over the entire length of the shoot, because it is recognised that the shoot both perceives and responds to gravity along its entire length (Firn and Digby 1980; Hashiguchi et al. 2013; Moulia and Fournier 2009). In the case of inflorescence stems of Arabidopsis, the gravitropic response is spatially restricted by lignification though perception can occur throughout their length (Okamoto et al. 2015; Weise et al. 2000).

Recently, Bastien et al. (2013, 2014) have determined movement along the entire length of the shoot during a gravitropic response, and have proposed a new model for gravitropism, the graviproprioception (GP) model. This model is a revised model of Sachs’ sine law, obtained by incorporating curvature, *C*, as a second explanatory variable to the sine law. The sine law was first proposed by Sachs (1879), and has been widely used for over 100 years. It is solely dependent on a single variable, the angle of deflection to the vertical, *A*, and states that the magnitude of the gravitropic response is proportional to sin*A* (Firn and Digby 1980; Moulia and Fournier 2009; Sachs 1879). However, the sine law has a deficiency, as it does not explain the observation that whilst the deflected organ undergoes curvature to bring it back to a vertical position, parts of the organ begin to grow straight before the vertical is achieved. This response is called autostraightening or autotropism (Firn and Digby 1979; Stanković et al. 1998; Tarui and Iino 1997). To correct this deficiency a new component, *C*, has been added to the sine law in the GP model, *C* being a measure to show how fast the tangent line of a curve changes its direction along a curve. Therefore, *C* is defined as d*A*/d*s*, where *s* is the curvilinear length or arc length of the curve. *C* of a straight line is zero; the larger the magnitude of *C*, the more pronounced is the bending of the curve. If a curve with a positive *C* bends in one direction, another curve with a negative *C* bends in the opposite direction. Any curve can be locally approximated by a unique circle. Thus, in another definition, *C* of the curve is defined to be the reciprocal of the radius of the circle. According to either definition, the unit of *C* is m^−1^.

As well as geometric characteristics of hypocotyls during gravitropism, we examined the expression of the *Indole-3-Acetic Acid 19* (*IAA19*) gene along the length of the hypocotyl, using bioluminescence imaging of hypocotyls harboring an *IAA19* promoter-driven luciferase reporter. IAA19 is one of the Auxin/IAA (Aux/IAA) auxin coreceptors, and is thought to function as a transcriptional repressor for auxin response factors (ARF; Guilfoyle 2015; Salehin et al. 2015), such as ARF5/MONOPTEROS (Krogan et al. 2014), ARF7/NON-PHOTOTROPIC4 (Tatematsu et al. 2004) and ARF19 (Lavenus et al. 2013). A dominant mutant of *IAA19, massugu2* (*msg2*), in which the IAA19 protein is stabilized due to an amino-acid substitution in its degron sequence, domain II, exhibits aberrant hypocotyl gravi- and phototropic responses (Tatematsu et al. 2004). Use of the promoter-β-glucuronidase (GUS) reporter fusion revealed differential expression of *IAA19* in gravi- and phototropic responses, with higher expression in the convex half of the hypocotyl (Kami et al. 2014; Saito et al. 2007). During a phototropic response, expression of *IAA19* is also positively regulated by basic Helix-Loop-Helix (bHLH)-type transcription factors, PHYTOCHROME INTERACTING FACTOR4 and 5 (Sun et al. 2013). *IAA19* is inducible by brassinosteroid (Nakamura et al. 2003) through an atypical bHLH-type transcription factor, BRASSINAZOLE-RESISTANT1 (Zhou et al. 2013), as well as by auxin (Lewis et al. 2013; Tatematsu et al. 2004). In fact, shoot gravitropism is, in part, negatively regulated by brassinosteroid (Nakamoto et al. 2006), which is achieved through expression of *Aux*/*IAA* genes including *IAA19* (Vandenbussche et al. 2012). Further, *IAA19* expression is positively correlated with hypocotyl growth in the shade-avoidance response (Pierik et al. 2009), and is inducible by humic substances (Trevisan et al. 2009).

Luciferase as a reporter is often used to monitor rapidly changing gene expression, e.g. as observed in circadian phenomena (Millar et al. 1992). Luciferase has an advantage over fluorescence-emitting proteins like green fluorescent protein (GFP), because it does not require light irradiation for excitation, and thus detection of luciferase activity does not affect the physiological state of plants, which are usually strongly light sensitive. Further, the fact that luciferase has a shorter half-life than GFP means that it provides a higher temporal resolution for responses (de Ruijter et al. 2003). Thus luciferase was used as a bioluminescence imaging probe to detect rapid movement of abiotic stress-induced systemic signals along leaves and stems, where its rate was determined to be 8.4 cm min^−1^ (Miller et al. 2009).

Here we used destabilized enhanced green-emitting luciferase (ELuc-PEST) as a bioluminescence imaging probe (Nakajima et al. 2010) to study the gravitropic response of etiolated Arabidopsis hypocotyls. The PEST element of the mouse ornithine decarboxylase, which is often used as a degron, was fused in-frame to the C-terminus of ELuc. ELuc is a luciferase of *Pyrearinus termitilluminans*, which is brighter than firefly luciferase due to higher expression level and protein stability. The half-life of ELuc-PEST is reported to be 3–4 h (Nakajima et al. 2010), while that of ELuc is 10 h (Yasunaga et al. 2015) in mammalian culture cells at 37 °C.

In this study, we measured three geometric parameters of hypocotyls, that is, *A, C*, and its partial derivative with respect to time, $\partial C/\partial t$, as well as promoter activity of *IAA19* as a function of time, *t*, and *s* during a gravitropic response, and constructed time–space maps of these parameters. We determined which model better explained hypocotyl gravitropism, comparing these *t–s* maps, and found that the hypocotyl gravitropic response was composed of multiple elements that are each determined by separate principles.

## Materials and methods

### Transgenic lines

The *ELuc-PEST* sequence was amplified by polymerase chain reaction (PCR) using a pair of oligonucleotides with the primer sequences, 5′-ATGGGATCCATGGAGAGAGAGAAGAACGTG-3′ and 5′-GACTCTAGACTCACACATTGATCCTAGCAGA-3′, and a plasmid, *pELuc(PEST)-test* (Toyobo) as a template. It was digested by BamHI and XbaI and cloned into *pART7* cloning vector (Gleave 1992). The *IAA19* promoter sequence was amplified by PCR using a pair of oligonucleotides with the primer sequences, 5′-ATGGAGCTCGCGGCCGCGTTCCTTCGCATCGGATTTGACGAAGATC-3′ and 5′-CATGAATTCGGGATCGATGTCGACTTCTTGAACTTCTTTTTTTCCTCTCACAAT-3′, and the genomic DNA of Arabidopsis (Col-0) as a template. The 3106-bp *IAA19* promoter fragment was cloned into EcoRI-BamHI site of the above-mentioned *pART7-ELuc-PEST*, and the resulting expression cassette containing a NotI site was recloned into a *pART27* binary vector (Gleave 1992) for *Agrobacterium*-mediated floral dip transformation (Clough and Bent 1998). All PCR was performed with Phusion DNA polymerase (Thermo Fisher Scientific). *pIAA19:ELuc-PEST* was introduced to the *msg2-1* mutant by crossing.

### Imaging

Bioluminescence imaging was performed using a horizontally placed microscope (MVX10, Olympus) with a 1× objective (MVPLAPO 1×, N.A. = 0.25) equipped with a cooled electron multiplying charge coupled device (EM-CCD) camera (ImagEM, Hamamatsu Photonics) with a filter wheel (Prior Scientific). Luminescence (Lumi) images were acquired at 1 × 1 binning of the 512 × 512 pixel array with an EM gain of 200 and exposure time of 30 or 60 s. Lumi intensity was 16 bit AD converted light intensity/pixel. Just before acquisition of a Lumi image, a bright-field image was obtained through a neutral density filter with an EM gain of 0 and exposure time of 0.3 s using dim green light obtained by filtering white light-emitting diode light through green plastic film. The whole imaging system was placed in a dark box (122 × 74 × 91 cm), which was proved to be light-tight because the level of background signal observed with the EM-CCD camera at the maximum EM gain was the same as that obtained when the shutter of the camera was closed. The imaging system, data acquisition and filter wheel controller were controlled by MetaMorph (v. 7.6.1.0, Molecular Devices).

**Fig. 1:**
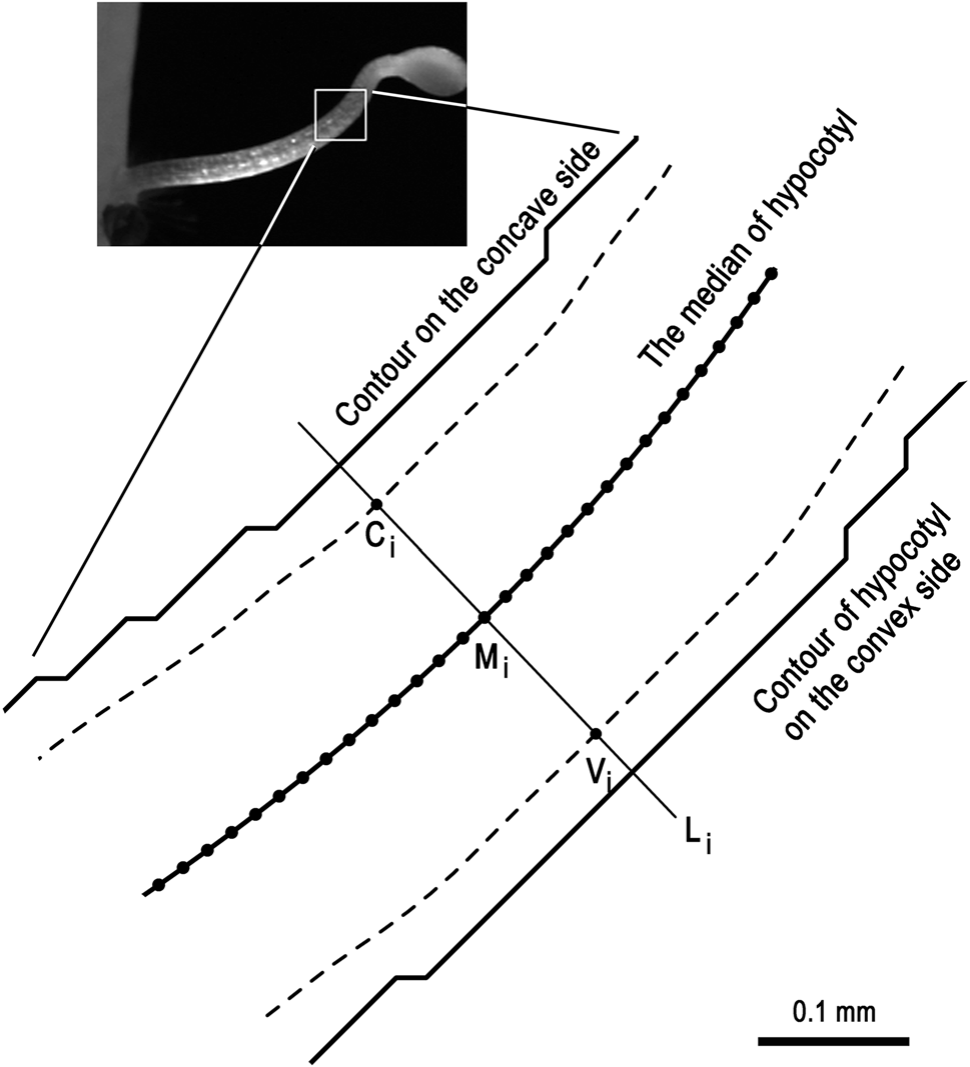
Schematic representation of measurement of luminescence intensity along the hypocotyl. The *bold lines* show the median of hypocotyl and the hypocotyl surface contours on the concave and convex sides. The broken lines are smoothed lines that are located 1/8 of the hypocotyl diameter inside from the contour lines. Points M_i_ are set on the median of the hypocotyl at 19.9-µm interval from the base to the tip. Line L_i_ is normal to the median at M_i_. C_i_ and V_i_ are intersection points between L_i_ and the smoothed, internal line on the concave side and the convex side, respectively. Luminescence intensity was measured at C_i_, M_i_ and V_i_, and is referred to as *L*_c_, *L*_m_ and *L*_v_, respectively. *Inset* shows a hypocotyl 1.36 h after turning to the horizontal in Fig. 2

**Fig. 2:**
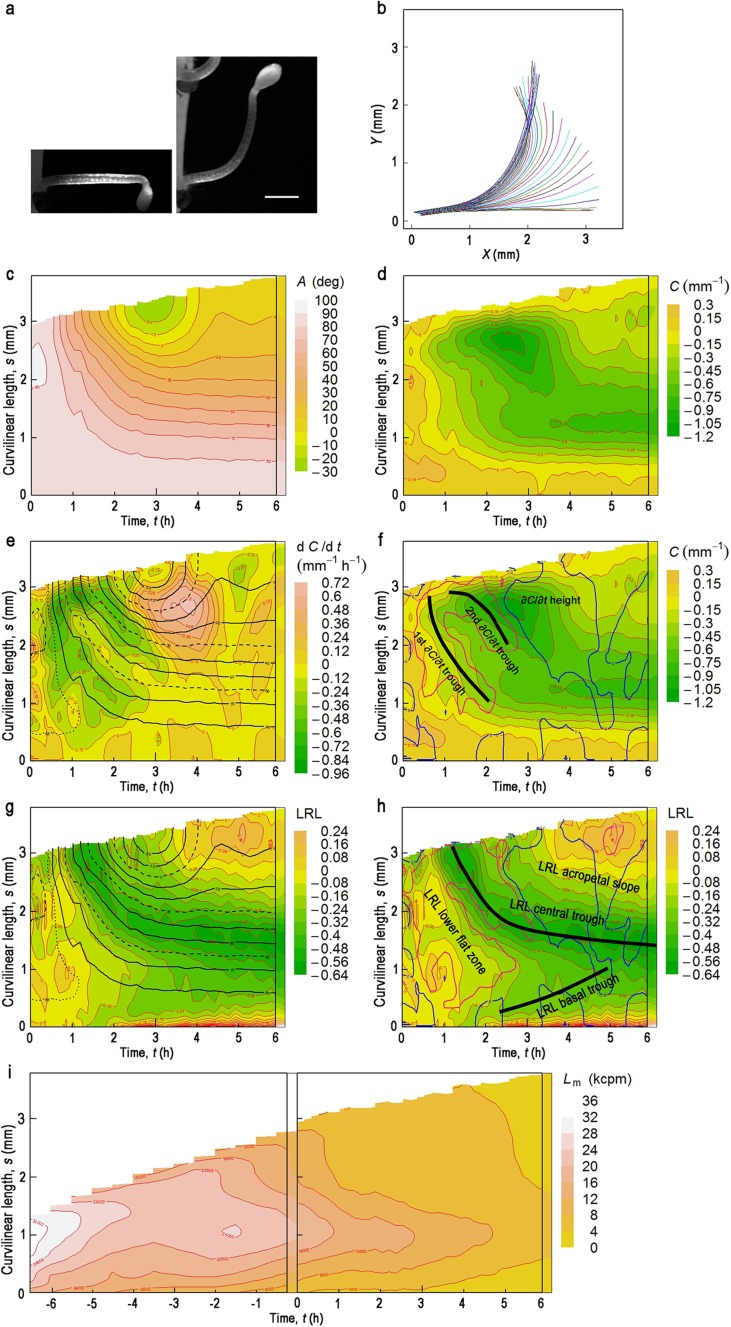
Gravitropic response of an etiolated hypocotyl harboring *pIAA19:ELuc-PEST*, with cotyledons facing downwards. Contour maps in (**c**–**h**) are drawn from the data shown in (**b**). **a** The examined hypocotyl at *t* = 0 (*left*) or 6.10 h (*right*) after turning to the horizontal in darkness. *Bar* = 1 mm. **b** Change in the median position of the hypocotyl after turning through 90°. Measurements were made at 0.170-h intervals over 6.10 h, in darkness. Successive positions of the median are shown in *different colors*, in the order of *black, red, green, blue, cyan*, and *magenta*. **c** A contour map of the deflection angle (*A*) in the *t–s* plane. **d** A contour map of curvature (*C*) in the *t–s* plane. **e** A contour map of $\partial C/\partial t$ in the *t–s* plane (Fig. S1) superimposed with the contour lines of *A* (**c**) at 10° intervals (*black solid line*). Contour lines of 0°, 30°, 60° and 90° are drawn as a broken line; that of 85° as a dotted line. **f** The *C* map with topographic characteristics of the $\partial C/\partial t$ map (Fig. 2e). *Red* and *blue lines* are the contour lines of $\partial C/\partial t$ = −0.3 and 0, respectively, from Fig. 2e. *Thick black lines* show troughs of $\partial C/\partial t$. **g** A contour map of the logarithm of the ratio of luminescence of the concave side to that of the convex side (log_10_(*L*_c_/*L*_v_); LRL) in the *t–s* plane, superimposed with contour lines of *A* in the same fashion as in (**e**). **h** The LRL map with topographic characteristics and contour lines of $\partial C/\partial t$ = −0.2 (*red*) and 0 (*blue*) from (**e**). *Thick black lines* show troughs of LRL. **i** A contour map of luminescence intensity along the median of hypocotyl (*L*_m_) in the *t–s* plane. At time 0, a hypocotyl was turned by 90°

Arabidopsis seeds were imbibed at 4 °C for a week, sown on 0.9% (w/w) agar medium containing 1% (w/w) sucrose and 1/2 MS salts (Murashige and Skoog 1962), and incubated at 23 °C under continuous white light for 24 h. Germinating seeds were chosen and placed on a flat bed of the same agar medium containing 200 µM luciferin in a 1-well coverglass chamber (5202-001, AGC Techno Glass), so that hypocotyls were able to grow freely in any direction without contact with agar surface (Figs. 1, 2a). After incubation at 23 °C for 36 h in the dark, the chamber was set on the imaging system for measurement, in darkness at ~23 °C.

### Image processing

From the bright-field image, hypocotyl contours were extracted by the consecutive use of “Make Binary”, “Find Edges” and “Skeletonize” functions of ImageJ. The median of the hypocotyl was obtained from the dataset of midpoints between contours on the convex side and the concave side of hypocotyl (Figs. 1, 2b) by principal curve analysis (Hastie and Stuetzle 1989) implemented in the pcurve package of R with degree of freedom (df) of 10. We then parameterized the median by the curvilinear length, *s*, from the base (*s* = 0) to the tip, every 19.9 μm. Partial derivative was obtained in R by fitting a cubic smoothing spline to the dataset, in which df was set to be half of the data size.

Lumi intensity (*L*) in the Lumi image was measured in cpm at a given point in ImageJ. *L* of the median (*L*_m_) was acquired at each parameterized point, M_i_ (Fig. 1). In order to determine *L* of the convex and concave sides of the hypocotyl (*L*_v_ and *L*_c_, respectively), contour lines which were located 1/8 of the diameter of hypocotyl inside from the hypocotyl surface were determined by principal curve analysis, in the same manner used to determine the median, except for the use of df of 60 (Fig. 1, broken line). Then, coordinates of two points, V_i_ and C_i_, were calculated, so that a line which was normal to the median at each parameterized point, M_i_, crossed the 1/8-inside contour line on the convex side and the concave side at V_i_ and C_i_, respectively (Fig. 1). *L*_v_ and *L*_c_ were measured at V_i_ and C_i_. *L*_m_, *L*_v_ and *L*_c_, which were functions of *s*, were next smoothed with cubic smoothing spline with df of a half of the data size, and the resultant smoothed data were shown as observed data in this study. All the calculation and drawing of contour maps were conducted in R (ver. 2.14.1; https://www.r-project.org/).

## Results

### Gravitropism in hypocotyls with downward-facing cotyledons

**Fig. 3:**
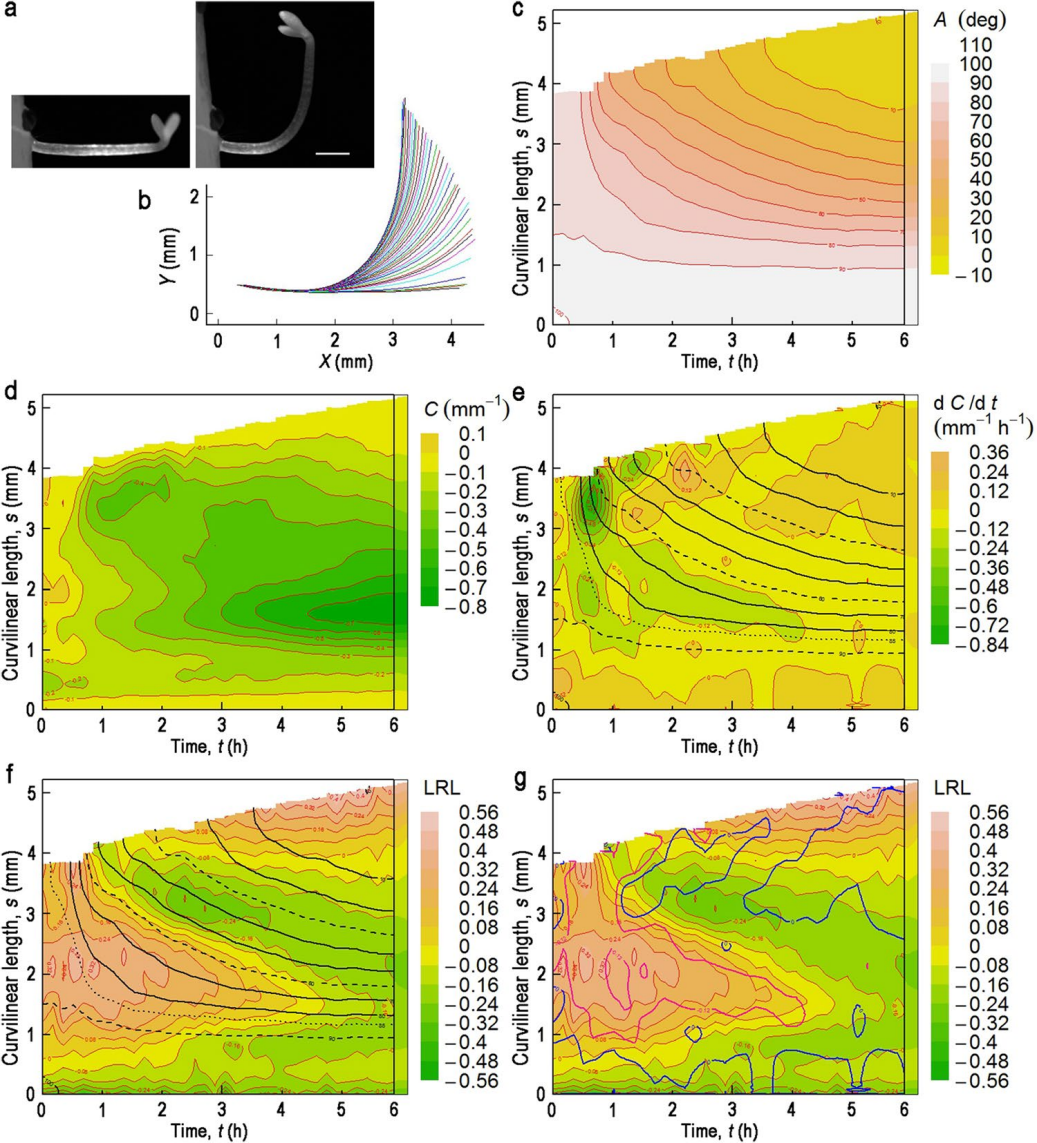
Gravitropic response of an etiolated hypocotyl harboring *pIAA19:ELuc-PEST*, with cotyledons facing upwards. Contour maps in (**c**–**g**) are drawn from the data shown in (**b**). **a** The examined hypocotyl at *t* = 0 (*left*) or 6.10 h (*right*) after turning to the horizontal in darkness. *Bar* = 1 mm. **b** Change in the median position of the hypocotyl after turning through 90° in darkness. For more details, see legend to Fig. 2b. **c**–**g** Contour maps of deflection angle (*A*) (**c**), curvature (*C*) (**d**), $\partial C/\partial t$ (**e**), and LRL (**f** and **g**) in the *t–s* plane. In **e** and **f**, contour lines of *A* are superimposed with 10° intervals (*black solid line*). Contour lines of 0°, 30°, 60° and 90° are drawn as a *broken line*; that of 85° as a *dotted line*. In **g**, contour lines of $\partial C/\partial t$ = −0.12 (*red*) and 0 (*blue*) are superimposed

We carried out a time-course study of the gravitropic response in etiolated Arabidopsis hypocotyls harboring *pIAA19:ELuc-PEST*. It should be noted that examined hypocotyls were grown without contact with agar medium in this study, so that they could move freely in any direction. In etiolated hypocotyls, cotyledon position influences the gravitropic and phototropic responses; in the gravitropic response, seedlings in which the cotyledons face downwards exhibit faster bending (Khurana et al. 1989). We therefore turned seedlings to the horizontal with the cotyledons facing downwards, and then captured bright-field and Lumi images at ~10-min intervals over ~6 h in the dark. Hypocotyls with partially open hooks were used for measurements as these gave the clearest images (Figs. 2a, 3a).

### Deflection angle, curvature and its partial derivative

The central axis of the hypocotyl, the median, was calculated from the bright-field images and was parameterized from the base (*s* = 0) to the tip every 19.9 μm (Figs. 1, 2a, b). The local deflection angle with respect to the vertical (*A*) and the corresponding local curvature (*C*) were thus obtained as functions of *s* and *t*, and are displayed as a contour map in the *t–s* plane (Fig. 2c, d, respectively). As illustrated in the time–space map of *A*(*s, t*) in Fig. 2c, the hypocotyl tip reached the vertical ~ 2 h after turning the hypocotyl to the horizontal. The tip continued to bend past the vertical, to below −20° at *t* = ~3 h; it then moved back to the vertical, after which it began to oscillate around the vertical position. As described above, *C* was defined as $\partial A\left( {s,t} \right)/\partial s$, where *s* is defined as being zero at the base of the hypocotyl and its value increases along the hypocotyl to the tip. Thus, after turning the hypocotyl by 90°, *C* was decreased to below zero (Fig. 2d).

The partial derivative of *C* with respect to *t* at position *s* ($\partial C\left( {s,t} \right)/\partial t$) is likely to be the most appropriate measure for comparison with any cellular activities (Bastien et al. 2013; Moulia and Fournier 2009; see below for more discussion). $\partial C\left( {s,t} \right)/\partial t$ was actually calculated from the predicted *C*(*s, t*) which was obtained by smoothing the observed *C*(*s, t*) (Fig. 2d) at each position *s*. $\partial C\left( {s,t} \right)/\partial t$ is illustrated as a contour map in Figs. S1, 2e. Graphically $\partial C\left( {s,t} \right)/\partial t$ is differentiation of *C*(*s, t*) in the direction parallel to the *t* axis. In Fig. 2f, the *C*(*s, t*) map has been superimposed on the contour lines of $\partial C\left( {s,t} \right)/\partial t$ to make clear relationship between *C* and $\partial C/\partial t$ and the topographic features of $\partial C/\partial t$. A single prominent depression of *C* appeared near the tip at *t* = ~2.5 h, and it then moved basipetally (Fig. 2d). Curvature was finally concentrated near the base at *s* = ~1.2 mm. However, when $\partial C/\partial t$ was examined (Figs. S1, 2e, f), we found that bending was, in fact, a two-step process: the first step, which is represented as the first $\partial C/\partial t$ trough in Fig. 2f, started to occur very early in the gravitropic response (~1/3 h after turning the hypocotyl horizontally), and was observed over almost the entire length of the hypocotyl. The second step, which is shown as the second $\partial C/\partial t$ trough in Fig. 2f, occurred later, and was restricted to a shorter section of the hypocotyl near the tip. We examined the gravitropic response independently in 8 hypocotyls. The two-step process was observed in 5 of the 8 hypocotyls, in two other cases the second trough was shallower than the first one (Fig. S2a), and in the final case the second trough appeared as just a small protrusion from the first one, near the tip (Fig. S2b). After curving to a maximum, the apical part of the hypocotyl started de-curving, represented in Fig. 2e as positive $\partial C/\partial t$ values. The decurving response or autostraightening occurred first near the tip, then migrated basipetally along the hypocotyl, but it persisted for longer than each of the curving responses. The positive $\partial C/\partial t$ region, which we name the $\partial C/\partial t$ height in Fig. 2f, is the region where autostraightening of the hypocotyl occurs.

Superimposing contour lines of *A* on the $\partial C/\partial t$ map (Fig. 2e) shows that the first trough aligns well with a contour line of *A* = 75°. The second trough, while not aligned as well as the first one, does align roughly with the 30° line. For each of the 8 measurements we determined *A* for which the $\partial C/\partial t$ troughs fitted with a resolution of 5°. As a result, the first trough was observed at *A* = 76° ± 4°, and the second one at 38° ± 7°. These results clearly indicate that during a gravitropic response, hypocotyls bend, responding locally to *A* along almost their entire length. The first trough was deepest in the apical region, and became shallower towards the basal region. However, the trough corresponded well to the same value of *A* over the entire length of the hypocotyl. These results suggest that although the magnitude of response differs, the threshold deflection angle to which hypocotyls respond does not change along the entire length of the hypocotyl.

$\partial C/\partial t$was calculated from the predicted *C*. In order to examine the accuracy of the prediction, we calculated the standard error of the estimate (SEE) between the predicted *C* and the observed *C*. The ratio of the SEE to the observed *C* is displayed as a time–space map on a logarithmic scale (Fig. S3). The SEE ratio was larger at *t* < ~3/4 h and near the base of hypocotyl (*s* < ~0.5 mm), where estimation of $\partial C/\partial t$ must be inaccurate.

### *IAA19* gene expression

To determine differential gene expression of *IAA19*, we measured Lumi intensity on the convex and concave sides of the hypocotyl (*L*_v_ and *L*_c_, respectively; Fig. 1), using the logarithm of the ratio of *L*_c_ to *L*_v_ (log_10_(*L*_c_/*L*_v_); LRL) as a parameter for differential expression (Fig. S4, Fig. 2g, h). We also measured Lumi intensity along the median of the hypocotyl (*L*_m_) for ~6 h before turning hypocotyls to the horizontal. Figure 2i (left and right) illustrates the *L*_m_(*s, t*) maps before and after turning the hypocotyl, respectively. Initially after germination hypocotyls emitted strong Lumi in the apical region, but as seedlings grew in the dark, *L*_m_ gradually decreased, and the region of strongest Lumi moved basipetally, finally reaching a position of *s* = ~1.2 mm. This pattern of change in *L*_m_ was essentially not affected by turning of the hypocotyl through 90°. The distribution of Lumi along the hypocotyl is in good agreement with the expression pattern previously reported using a GUS reporter driven by the same *IAA19* promoter (Kami et al. 2014).

After turning the hypocotyl, LRL is also illustrated as a time–space map in Fig. 2g, and its topographic features are indicated in Fig. 2h. The reduction in LRL, that represents an increase in *IAA19* expression of the convex side relative to that of the concave side, first appeared in the apical part ~1.5 h after turning to the horizontal, and then migrated basipetally, forming the LRL central trough. This was formed due to both an increase in *L*_v_ and a decrease in *L*_c_ along the hypocotyl (Fig. S5). Superposition of contour lines of *A* on the LRL map shows that the central trough aligns well with a contour line of 44° ± 4° (n = 8). This means that the LRL central trough is completely separated from the first $\partial C/\partial t$ trough, as the first $\partial C/\partial t$ trough is located in the LRL lower flat zone (Fig. 2h). In contrast, the second $\partial C/\partial t$ trough almost overlaps the LRL central trough. In one of the hypocotyls, where the second $\partial C/\partial t$ trough was just a small protrusion from the first $\partial C/\partial t$ trough (Fig. S2b), the LRL central trough coincided with the $\partial C/\partial t$ height. These observations indicate that differential expression of *IAA19* is not a factor in the first curving response. They also suggest that the transcriptional control of differential *IAA19* expression and the second curving response may overlap to some extent.

It is noteworthy that most part of the $\partial C/\partial t$ height was located where LRL was below zero (Fig. 2h). Curving and decurving are likely to occur due to differential elongation between the convex and concave sides of the hypocotyl (Moulia and Fournier 2009). If we assume that LRL reflects the formation of an auxin concentration gradient along the transverse axis of hypocotyl, this result means that hypocotyls start to decurve, i.e. the concave side of the hypocotyl starts to grow faster than the convex side, even when the auxin content of the convex side is higher than that of the concave side.

### Gravitropism of hypocotyls with upward-facing cotyledons

The same analyses as described above were conducted with hypocotyls in which the cotyledons faced upwards (Fig. 3a). The rate of bending was slower than that of hypocotyls with downward-facing cotyledons (Fig. 3b, c). Almost no overshooting was observed in the tip region during the 6-h period of measurement. In fact, the lowest *A* observed in the 6-h time period was 8° ± 7° (n = 3) in hypocotyls with upward-facing cotyledons, while it was −10° ± 10° (n = 8) in those with downward-facing cotyledons. The magnitude of *C* was less, particularly in the tip region (Fig. 3d). The depth of the $\partial C/\partial t$ depression was as large as that observed in hypocotyls with downward-facing cotyledons (Fig. 3e). However, the second $\partial C/\partial t$ trough was less marked, and only a small additional depression, rather than a trough, was observed near the tip. The first trough aligns with a contour line of *A* = 73° ± 3° (n = 3), and a small additional depression is located at 45° ± 5°, which is essentially the same as that found in hypocotyls with downward-facing cotyledons (*P* = 0.24 and 0.13, respectively, in *t*-test). Hypocotyls with upward-facing cotyledons also showed decurving as indicated by a positive $\partial C/\partial t$, but the magnitude was smaller.

At *t* = ~0, LRL was greater than zero (Fig. 3f) in contrast to negative LRL values in hypocotyls with downward-facing cotyledons (Fig. 2g). This is in line with our previous finding, using a *GUS* reporter gene, that higher expression of *IAA19* was observed on the cotyledon-attachment side of the hypocotyl (Kami et al. 2014). The LRL central trough was shallower than that in hypocotyls with downward-facing cotyledons (Fig. 3f). However, it almost fits to a contour line of *A* = 40° ± 5° (n = 3); thus the relative relationship between LRL and $\partial C/\partial t$ seems to be essentially the same between the two hypocotyl orientations.

### The sine-law model vs. the graviproprioception model

**Fig. 4:**
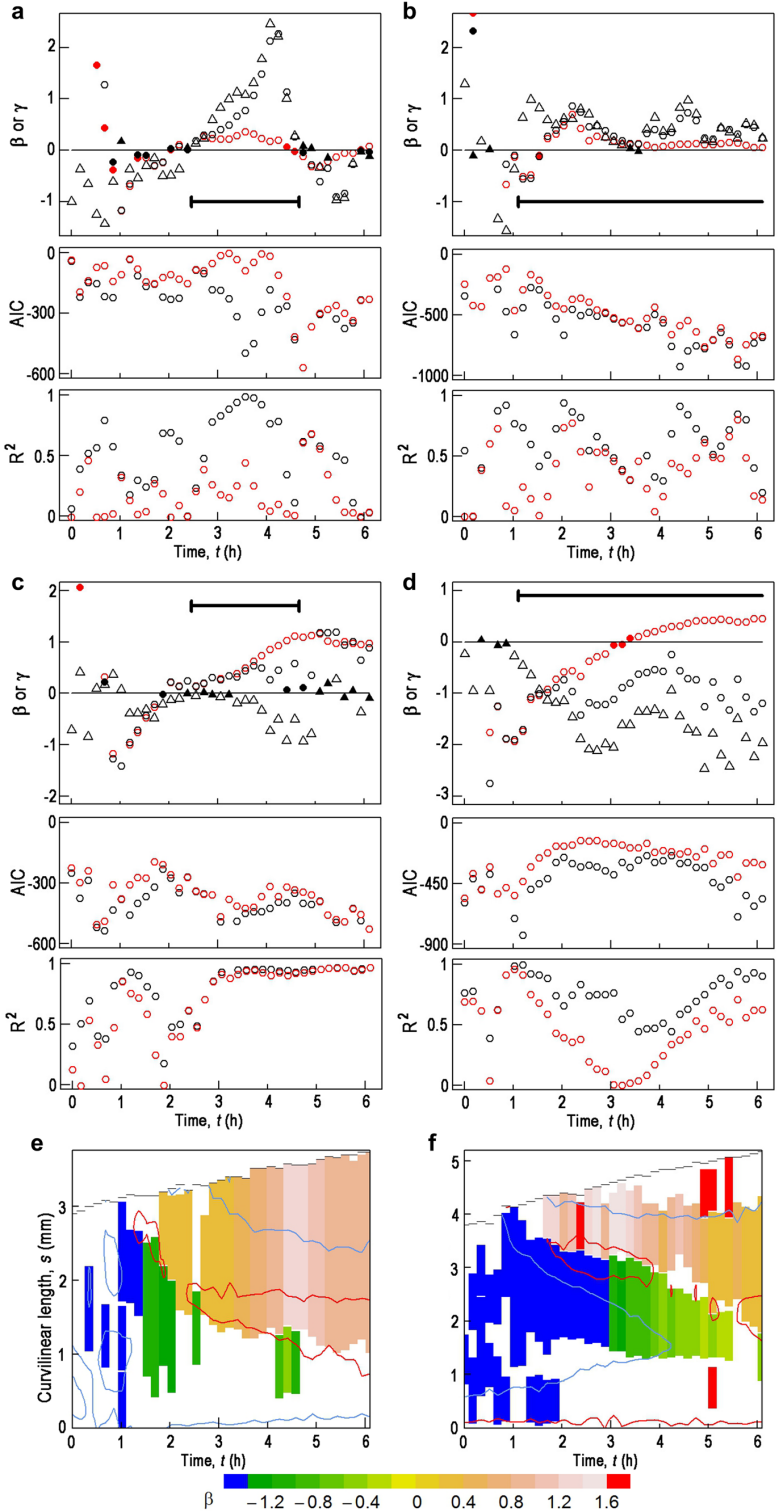
Parameters of the graviproprioception (GP) model and the sine-law model estimated for the hypocotyls in Figs. 2, 3 at each observed time point after turning through 90°. **a, b** Parameters estimated for $\partial C/\partial t$ from hypocotyls with downward (**a**) and upward (**b**)-facing cotyledons, respectively. In the top panels, for the GP model, β = *black circle* and γ = *black triangle*; for the sine-law model, β = *red circle. Solid symbols* mean that their values are not significantly different from 0 (*P* > 0.05). Thick horizontal lines indicate the time period when autostraightening occurred in Figs. 2e, 3e. In the middle and bottom panels, the Akaike information criterion (AIC) and the adjusted R^2^ are shown, respectively, for the GP model (*black*) and the sine-law model (*red*). Parameters were estimated for the apical portions of hypocotyls of which *s* was longer than 1.14 mm for **a**, and 1.33 mm for **b**. Some of the values of β and γ at *t* < 0.7 h fall outside of the area of these plots, and thus are shown in Fig. S6. **c, d** Parameters estimated for LRL from the same hypocotyls as in **a** and **b**, respectively. LRL was modeled after the GP and the sine-law models, and parameters are shown as in (**a, b)**. For more details, see legend to **a** and **b**. Some of the values of β and γ at *t* < 0.9 h fall outside of the area of these plots, and thus are shown in Fig. S7. **e, f** A time–space map of β for the sine-law model for LRL from the same hypocotyls as in **a** and **b**, respectively. Segments of hypocotyls which were longer than 0.76 mm, and in which their LRL followed the sine-law model with R^2^ > 0.95, are shown as a *t*‒*s* map (see Fig. S8 for an example). Such segments are colored according to β as shown in the color key, which is common to (**e**) and (**f**). These maps are superimposed with contour lines of LRL = −0.48 (*red*) and 0 (*light blue*) in (**e**), and LRL = −0.225 (*red*) and 0 (*light blue*) in (**f**), respectively

Recently Bastien et al. (2013) have proposed the GP model for gravitropism, which is defined as:

1 $$\partial C\left( {s,t} \right)/\partial t~ = - \beta {\text{ sin}}A\left( {s,t} \right) - \gamma C\left( {s,t} \right),$$

where β >0 and γ >0. The second term on the right-hand side is a correction term for the sine law; thus Eq. 1 will describe the sine-law model when γ is zero. To determine which model would best explain our observed data we conducted multiple linear regression analysis along *s* at each time point for ~6 h after turning hypocotyls through 90° (Fig. 4). By multiple linear regression, it was examined whether $\partial C/\partial t$ was linearly correlated with sin*A* and *C* at a given time, *t*, as described in Eq. 1. For hypocotyls with downward-facing cotyledons, positive values for β and γ were obtained with the GP model during the time when autostraightening was observed (Fig. 4a (top), S6). Although positive values for β were also obtained for the sine-law model over the same time period, the GP model was judged to better explain our data since it gave a lower Akaike information criterion (AIC; Fig. 4a (middle)), which is a measure of the relative quality of statistical models (Konishi and Kitagawa 2008; Kubo 2012). Further, the GP model also gave a higher adjusted R^2^ (Fig. 4a (bottom)), which is a measure to show how well the observed data fit Eq. 1. Essentially the same conclusion was reached for gravitropism of hypocotyls with upward-facing cotyledons (Fig. 4b, S6).

The GP model and the sine-law model have been figured out to explain $\partial C/\partial t$ (Bastien et al. 2013; Moulia and Fournier 2009.) We then examined which model best described LRL. Figures 4c, d and S7 show that with the GP model, values of β and γ were largely negative or zero, whereas the sine-law model gave positive values for β at *t* > ~2.5 and ~3.5 h for hypocotyls with downward- and upward-facing cotyledons, respectively, indicating that LRL follows the sine-law model in the later phases of gravitropism. Next, we used linear regression analysis between LRL and sin*A* at each time point to determine in which parts of the hypocotyl LRL followed the sine-law model (Fig. S8). In Fig. 4e, f those segments of the hypocotyl that followed the sine law are displayed in a time–space map, and are colored according to the values of β. The sine-law model was valid in almost entire (Fig. 4e) or a major part (Fig. 4f) of the LRL acropetal slope (Fig. 2h) for hypocotyls with downward- and upward-facing cotyledons, respectively. This also supports the above finding that LRL follows the sine law.

### Gravitropism of the *msg2* hypocotyl

**Fig. 5:**
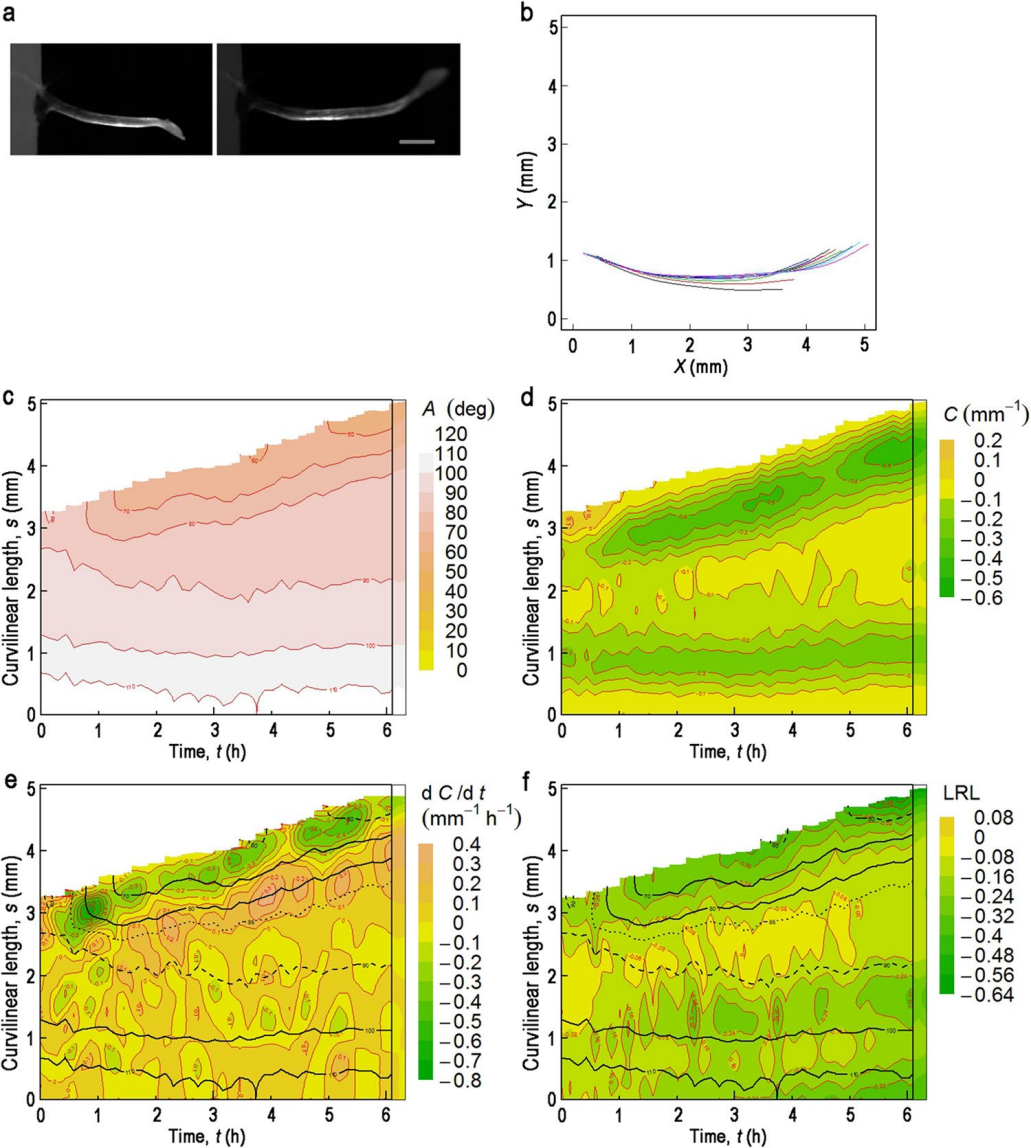
Gravitropic response of an etiolated *msg2-1* hypocotyl harboring *pIAA19:ELuc-PEST*. Contour maps are drawn from the data obtained at 0.144-h intervals over a period of 6.33 h after turning through 90° in darkness. **a** The examined hypocotyl at *t* = 0 (*left*) or 6.33 h (*right*) after turning to the horizontal in darkness. *Bar* = 1 mm. **b** Change in the median position of the hypocotyl after turning through 90° at 0.575-h intervals over 6.33 h. Successive positions of the median are shown in *different colors*, in the order of *black, red, green, blue, cyan*, and *magenta*. **c‒f** Contour maps of deflection angle (*A*) (**c**), curvature (*C*) (**d**), $\partial C/\partial t$ (**e**), and LRL (**f**) in the *t*–*s* plane. In **e** and **f**, contour lines of *A* are superimposed: contour lines of 60° and 90° are drawn as *broken lines*; 85° as a *dotted line*, and the other contour lines from 70° to 110° as *black solid lines*

The above analysis was applied to *msg2-1* hypocotyls; these exhibit much reduced gravitropism (Fig. 5a–c; Tatematsu et al. 2004), despite showing no significant defects in elongation rate (Fig. S9). After turning through 90°, depression of *C* appeared in the tip region (Fig. 5d), and this was as large as that in wild-type hypocotyls with upward-facing cotyledons (Fig. 3d). However, in contrast to the wild type, this depression did not appear in the central part of the hypocotyl, but remained in the tip region, where its magnitude increased slightly. We also found that in *msg2-1* depression of LRL remained consistently in the tip region, with slightly increasing magnitude, and that this was not observed in the central region (Fig. 5f). Contour lines of LRL in the tip region almost aligned with those of *A* (Fig. 5c, f). These observations suggest that in *msg2-1* only the tip region is able to respond to the gravity. It is also interesting that the peak of *L*_m_, which in the wild type moved basipetally as germinating seedlings grew (Fig. 2i), remained in the tip region in *msg2-1* (Fig. S10). In summary, the response of *msg2-1* hypocotyls is qualitatively different from that of the wild type, both for bending and *IAA19* expression, which agrees well with the gain-of-function nature of the *msg2* mutation (Tatematsu et al. 2004).

## Discussion

In this study we took regular measurements along the entire length of a hypocotyl for the first 6 h after turning through 90° to the horizontal, and then obtained *A, C*, and $\partial C/\partial t$, as a function of *t* and *s*. As a result, we found that the gravitropic response was composed of two consecutive curving responses and a subsequent de-curving response. The first curving response, represented as the first $\partial C/\partial t$ trough in the time–space map (Fig. 2f), is more robust than the second one in both space and time. It occurs early after turning the hypocotyl, and ends when each section of the hypocotyl reaches ~60° of *A* (Fig. 2e). Although in the time–space map the first curving response appears to migrate basipetally (Fig. 2e), it does not actually propagate downward. In fact it ends earlier in the more apical part because, due to the lever-arm effect (Moulia and Fournier 2009) the apical part reaches the critical 60° earlier than the basal parts. The lever-arm effect arises due to the fact that *A* of the apical part is an integral of *C* from the base (*s* = 0) to the apical part. Consequently *A* of the apical part decreases more quickly than that of the more basal part, and the change in curvature in the apical part continues for a short period of time, while in the more basal part it persists for a longer period of time. These results confirm well-known findings that in shoot gravitropism each shoot element perceives, and responds to, the gravity stimulus almost independently (Firn and Digby 1980; Hashiguchi et al. 2013; Moulia and Fournier 2009; Weise et al. 2000). The maximum of the first curving response that is represented by the first $\partial C/\partial t$ trough (Fig. 2f) aligns well with a contour line of *A* = 76° in the time–space map (Fig. 2e). This indicates that, whatever the functional relationship is between *A* and $\partial C/\partial t$, the first curving response is solely dependent on *A*. The most likely candidate for this function is the sine function, as stated by the sine law (Sachs 1879). Our model-selection approach did not indicate a sinusoidal relationship (Fig. 4a, b). However, this is probably because the resolution for model selection is too low due to smaller changes in *A* during the time period (*t* < ~1 h) when only the first curving response is observed.

The second curving response, represented by the second $\partial C/\partial t$ trough in the time–space map (Fig. 2f), is more variable than the first one. The length of the trough was more variable, indeed sometimes it did not form a trough, just being detected as a hole (Fig. 3e). Further, the second $\partial C/\partial t$ trough did not align with contour lines of *A* as well as the first one did (Fig. 2e), leading us to conclude that it may not be dependent solely on *A*, and that the second curving response is more complex than the first one.

A decurving or autostraightening response is represented by the $\partial C/\partial t$ height in the time–space map of $\partial C/\partial t$ (Fig. 2f). The shape and position of the $\partial C/\partial t$ height clearly show that the decurving response is not solely dependent on *A*. In fact, over the time period when a decurving response was observed, $\partial C/\partial t$ was better described by the GP model (Fig. 4a, b), where *C* is the other explanatory variable besides *A* (Eq. 1). Comparison between the *C* and $\partial C/\partial t$ maps (Fig. 2f) also indicates that a decurving response does not have a threshold with respect to *C*, as was proposed by Bastien et al. (2013).

We also determined LRL, a measure for differential promoter activity of *IAA19*, as a function of *t* and *s* during a gravitropic response. The largest decrease in LRL occurred along a contour line of *A* = 44° (Fig. 2g). LRL was well described by the sine-law model (Fig. 4c, d). If we assume that LRL reflects distribution of auxin in hypocotyls, these facts suggest that a gradient of auxin concentration is formed according to the sine law, which then results in expression of other auxin-induced genes. Comparison between the LRL and $\partial C/\partial t$ maps shows no direct relationship between LRL and a decurving response (Fig. 2h), suggesting that there is no significant involvement of auxin in this response. Haga and Iino (2006) report that auxin distribution is similar between the lower and upper flanks of pea epicotyls during autostraightening. Thus, they conclude that autostraightening occurs independently of auxin. Although our data suggest a significant gradient of auxin along the transverse axis of the hypocotyl, our conclusion is the same as theirs. Recently Okamoto et al. (2015) have proposed that the long actin filaments in elongating fibre cells act as a bending tensile sensor to perceive the posture of bending organs and trigger the straightening system.

If we assume that the first curving response is induced by differential accumulation of auxin, it is interesting that the LRL central trough, along *A* = 44°, was completely separated from the first $\partial C/\partial t$ trough, along *A* = 76°, since gene expression of *IAA19* has been shown to be induced by exogenously added auxin in a concentration-dependent manner (Tatematsu et al. 2004). This may be related to the observation that the affinity of IAA19 for TIR1/AFB auxin coreceptors and auxin is one of the lowest among Aux/IAA proteins expressed in the hypocotyl (Havens et al. 2012; Shimizu-Mitao and Kakimoto 2014; for expression in hypocotyl, see http://bar.utoronto.ca/efp/cgi-bin/efpWeb.cgi). According to Shimizu-Mitao and Kakimoto (2014), in yeast cells 0.26 µM IAA is necessary for 50% degradation of IAA7 through the TIR1-dependent pathway, and of all the Aux/IAAs expressed in hypocotyls, IAA7 is one of the most sensitive for auxin-dependent degradation. In contrast, 0.71 µM IAA is needed for IAA19 degradation. Because auxin-inducible IAA19 is a transcriptional repressor for ARF5 and ARF7, expression of *IAA19* is likely to be self-regulated through the ARF5/ARF7-IAA19 module (Krogan et al. 2014; Tatematsu et al. 2004). Therefore, it may be possible that the first curving response is regulated by the Aux/IAAs with a high affinity to auxin such as IAA7, and that IAA19 with a lower affinity starts to be differentially expressed later in the gravitropic response, when more auxin is accumulated in the convex side of hypocotyl. It would be interesting to examine the expression pattern in *t*–*s* space of high-affinity Aux/IAA proteins expressed in the hypocotyl, such as IAA7 and IAA4, during a gravitropic response.

In the present study, we showed that differential promoter activity of *IAA19* (LRL) correlated with *A* in the *t*–*s* plane with no discernable time lag between them. This is rather surprising because a certain period of time is required for the reporter gene to produce the ELuc-PEST protein, after its transcription. In this connection, a study of the wound-inducible Zat12 gene is worth-noting: Lumi of Arabidopsis plants harboring the Zat12 promoter fusion to luciferase increased significantly within 1 min after wounding (Miller et al. 2009), indicating that very little time is needed for synthesis of an optically detectable amount of luciferase. Here we measured hypocotyl images at ~10-min intervals, so we were unable to detect time lags less than 10 min.

In the Introduction, we noted that the partial derivative of *C* with respect to *t*, $\partial C/\partial t$, was the function to be analyzed during the gravitropic response. However, $\partial C/\partial t$ is a partial derivative, holding *s* constant, and we are, in fact, interested in *C* change of every elemental portion of hypocotyl, of which *s* increases over time. So, it is the material derivative of *C, DC*(*s, t*)/*Dt*, that should be considered in tropic responses of growing organs such as a hypocotyl (Bastien et al. 2014; Moulia and Fournier 2009). *DC*/*Dt* is a derivative of *C*, in which local growth rate of the hypocotyl is taken into account. Our attempt to determine local growth rate by tracing the position of the anticlinal cell wall of epidermal cells scattered along the hypocotyl was unsuccessful because growth axis of the measured hypocotyl was not always precisely on a focal plane during ~6-h-long measurements. Therefore, results of this study should be regarded as a first approximation for the gravitropic response.

Both gravitropic and phototropic responses are asymmetric with respect to the position of hook structure in hypocotyls (Khurana et al. 1989) and epicotyls (Kuhn and Galston 1992). They tend to bend more easily to the side bearing the convex portion of the hook than to the side bearing the concave portion of the hook. This asymmetry has been mostly studied in phototropism. Hypocotyls of the *pin-formed3* (*pin3*) *pin7* double mutants show phototropic defects only when the side bearing the concave part of the hook is irradiated with unilateral blue light (Haga and Sakai 2013); PIN3 and 7 are auxin efflux facilitators, which are likely to be involved in lateral transport of auxin (Adamowski and Friml 2015; Spalding 2013). As described above, the expression pattern of *IAA19* suggests that a higher level of auxin will have accumulated in the side of the hypocotyl under the concave portion of the hook before the application of a gravi- or a photostimulus, leading to a bending towards the convex side. Our findings therefore suggest that a higher amount of auxin must be transported laterally to generate the gradient of auxin concentration that is necessary for bending to the side bearing the concave part of the hook. Requirement of higher capacity for lateral auxin transport may suppress bending rate and overshooting in gravitropic response of hypocotyls with upward-facing cotyledons. Khurana et al. (1989), who first reported this asymmetry, speculated that the transmission or modulation of some signal along the hypocotyl, of which rates or extents were dependent on cotyledon position, resulted in asymmetric bending. Our and other observations described above suggest that the signal is most likely to be auxin.

In conclusion, we show here that gravitropic response of hypocotyl is composed of multiple elements. The first curving response is supposed to follow the sine law, because it occurs along the constant local deflection angle, *A*. Subsequent autostraightening follows the GP model, and is dependent on *A* and *C*. On the other hand, differential expression of *IAA19* follows the sine law even when hypocotyls show autostraightening. These results suggest that the gravitropic response follows the sine law until formation of the auxin concentration gradient, and then the proprioception mechanism that perceives *C* modulates output of the auxin-induced bending step.

## Electronic supplementary material

Below is the link to the electronic supplementary material.

Supplementary material 1 (PPTX 25437 KB)
