# Correction to: Space–time analysis of gravitropism in etiolated Arabidopsis hypocotyls using bioluminescence imaging of the *IAA19* promoter fusion with a destabilized luciferase reporter

**DOI:** 10.1007/s10265-018-1055-4

**Published:** 2018-07-18

**Authors:** Kotaro T. Yamamoto, Masaaki K. Watahiki, Jun Matsuzaki, Soichirou Satoh, Hisayo Shimizu

**Affiliations:** 1 Division of Biological Sciences, Faculty of Science, Hokkaido University, Sapporo, 060-0810 Japan; 2 Biosystems Science Course, Graduate School of Life Science, Hokkaido University, Sapporo, 060-0810 Japan; 3 Present Address: Center for Sustainable Resource Science, RIKEN, Tsurumi-ku, Yokohama, 230-0045 Japan; 4 Present Address: Graduate School of Life and Environmental Sciences, Kyoto Prefecture University, Sakyo-ku, Kyoto, 606-8522 Japan

## Correction to: J Plant Res (2017) 130:765–777 10.1007/s10265-017-0932-6

The article Space–time analysis of gravitropism in etiolated Arabidopsis hypocotyls using bioluminescence imaging of the *IAA19* promoter fusion with a destabilized luciferase reporter, written by Kotaro T. Yamamoto, Masaaki K. Watahiki, Jun Matsuzaki, Soichirou Satoh and Hisayo Shimizu, was originally published electronically on the publisher’s internet portal (currently SpringerLink) on 10 April 2017 without open access.

With the author(s)’ decision to opt for Open Choice the copyright of the article changed on 16 July 2018 to © The Author(s) 2018 and the article is forthwith distributed under the terms of the Creative Commons Attribution 4.0 International License (http://creativecommons.org/licenses/by/4.0/), which permits use, duplication, adaptation, distribution and reproduction in any medium or format, as long as you give appropriate credit to the original author(s) and the source, provide a link to the Creative Commons license and indicate if changes were made.

