# Supplementary material for: Space–time analysis of gravitropism in etiolated Arabidopsis hypocotyls using bioluminescence imaging of the IAA19 promoter fusion with a destabilized luciferase reporter
Source: J Plant Res. 2017 Apr 10;130(4):765–77. doi: 10.1007/s10265-017-0932-6 (PMC6105228; doi:10.1007/s10265-017-0932-6)
Supplement: Supplementary file 1 — Supplementary material 1 (PPTX 25437 KB) [file 10265_2017_932_MOESM1_ESM.pptx]

## Slide 1
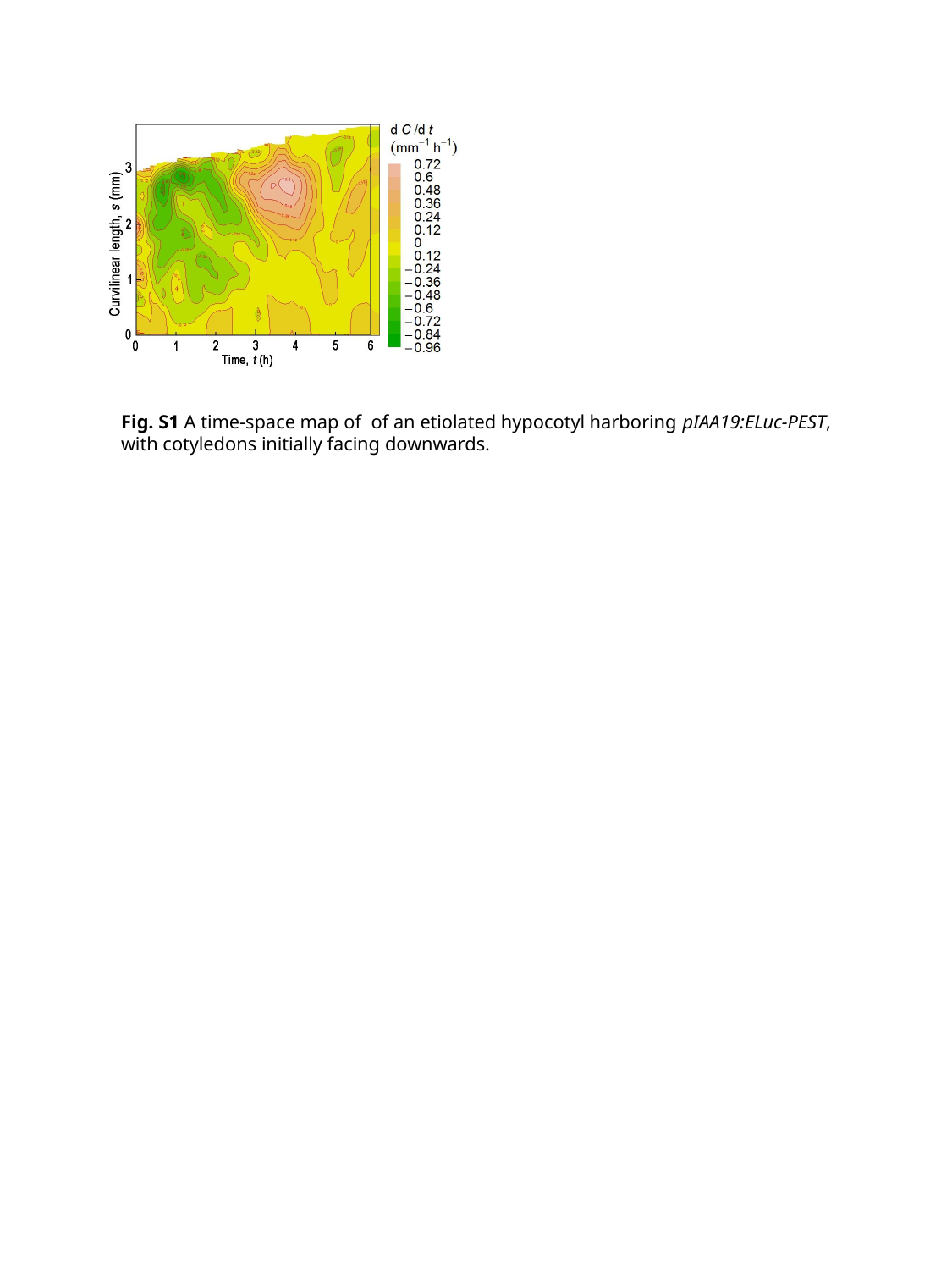

## Slide 2
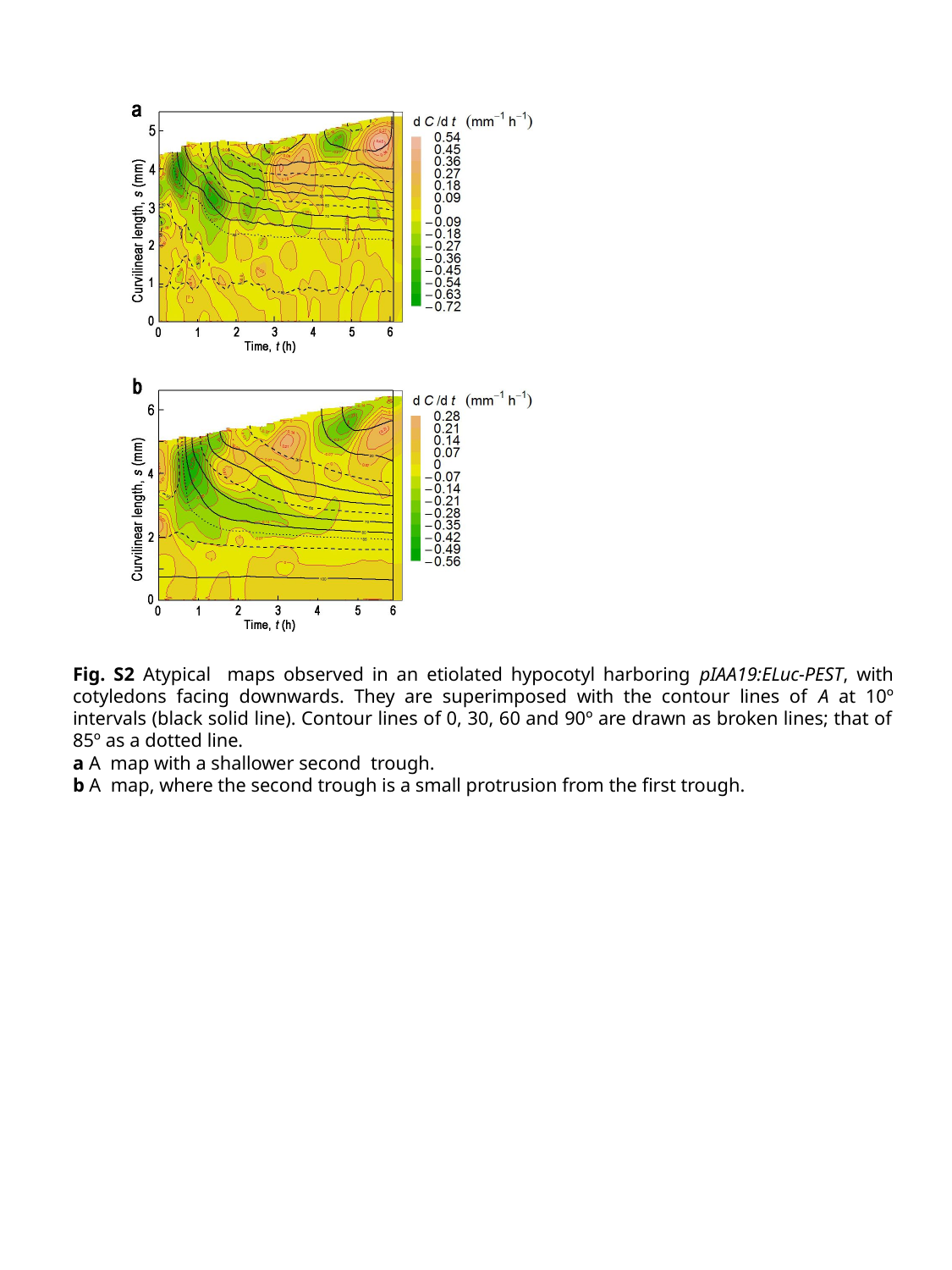

## Slide 3
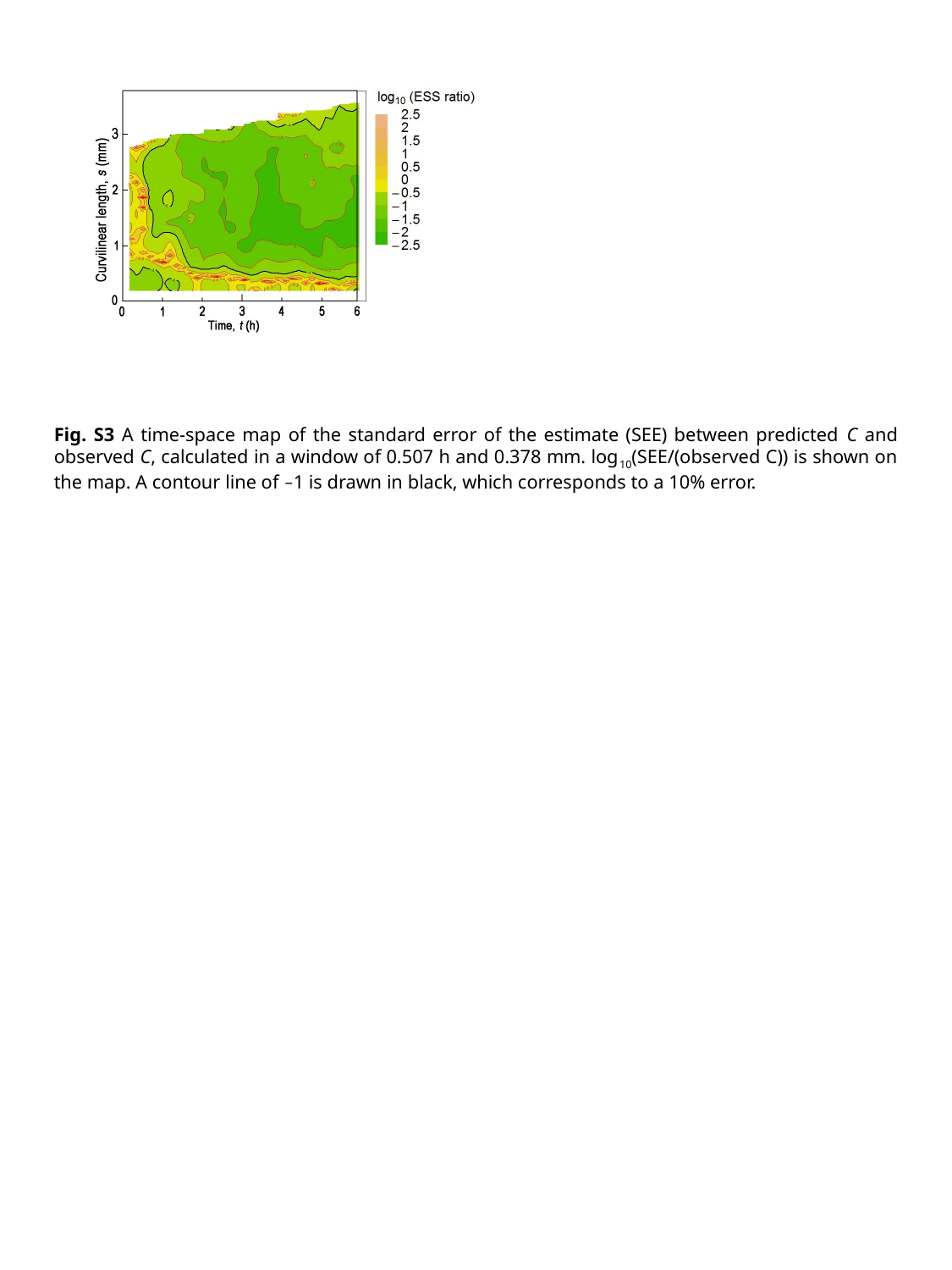

Fig. S3 A time-space map of the standard error of the estimate (SEE) between predicted C and observed C, calculated in a window of 0.507 h and 0.378 mm. log10(SEE/(observed C)) is shown on the map. A contour line of ‒1 is drawn in black, which corresponds to a 10% error.

## Slide 4
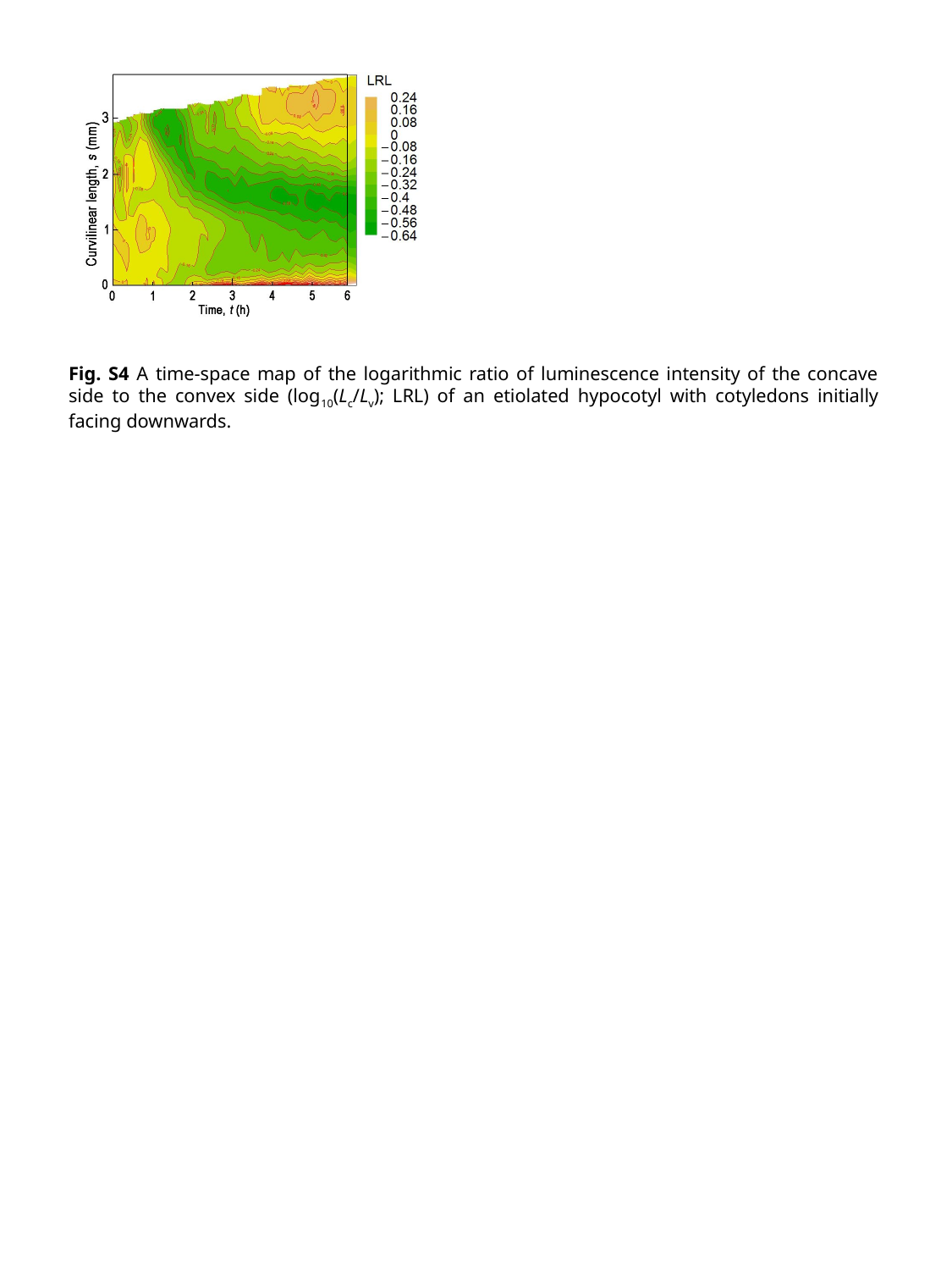

Fig. S4 A time-space map of the logarithmic ratio of luminescence intensity of the concave side to the convex side (log10(Lc/Lv); LRL) of an etiolated hypocotyl with cotyledons initially facing downwards.

## Slide 5
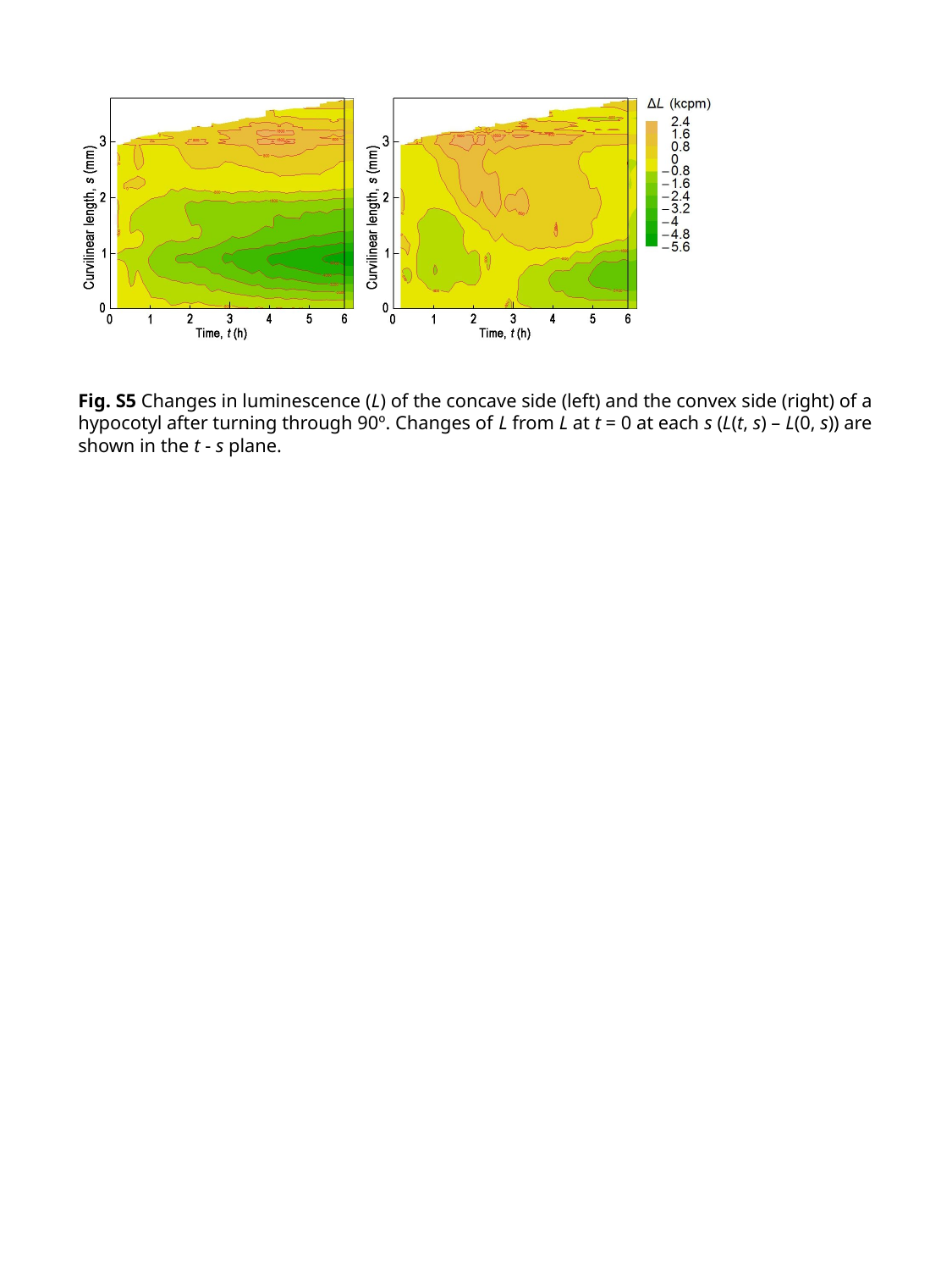

Fig. S5 Changes in luminescence (L) of the concave side (left) and the convex side (right) of a hypocotyl after turning through 90º. Changes of L from L at t = 0 at each s (L(t, s) – L(0, s)) are shown in the t - s plane.

## Slide 6
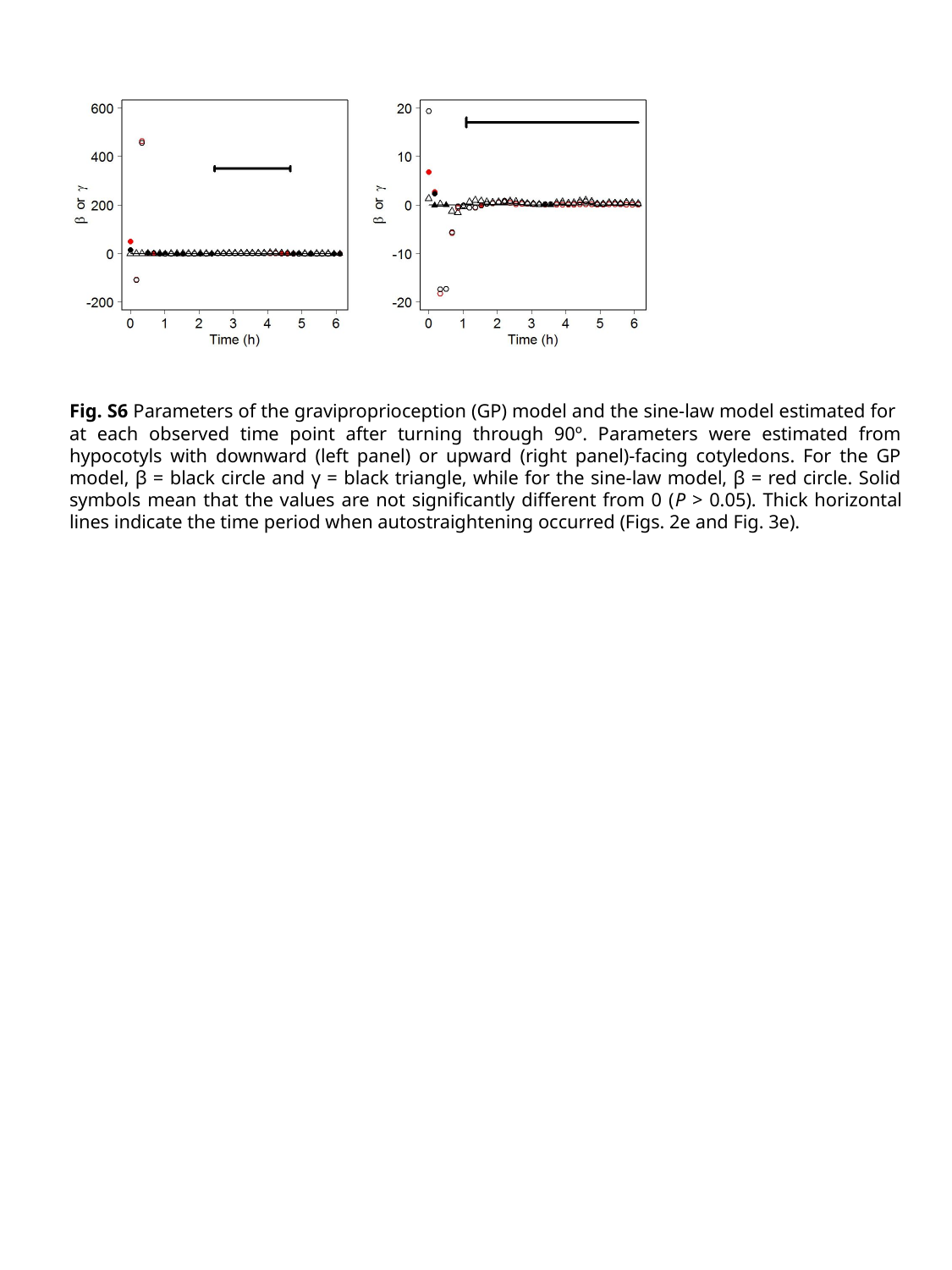

## Slide 7
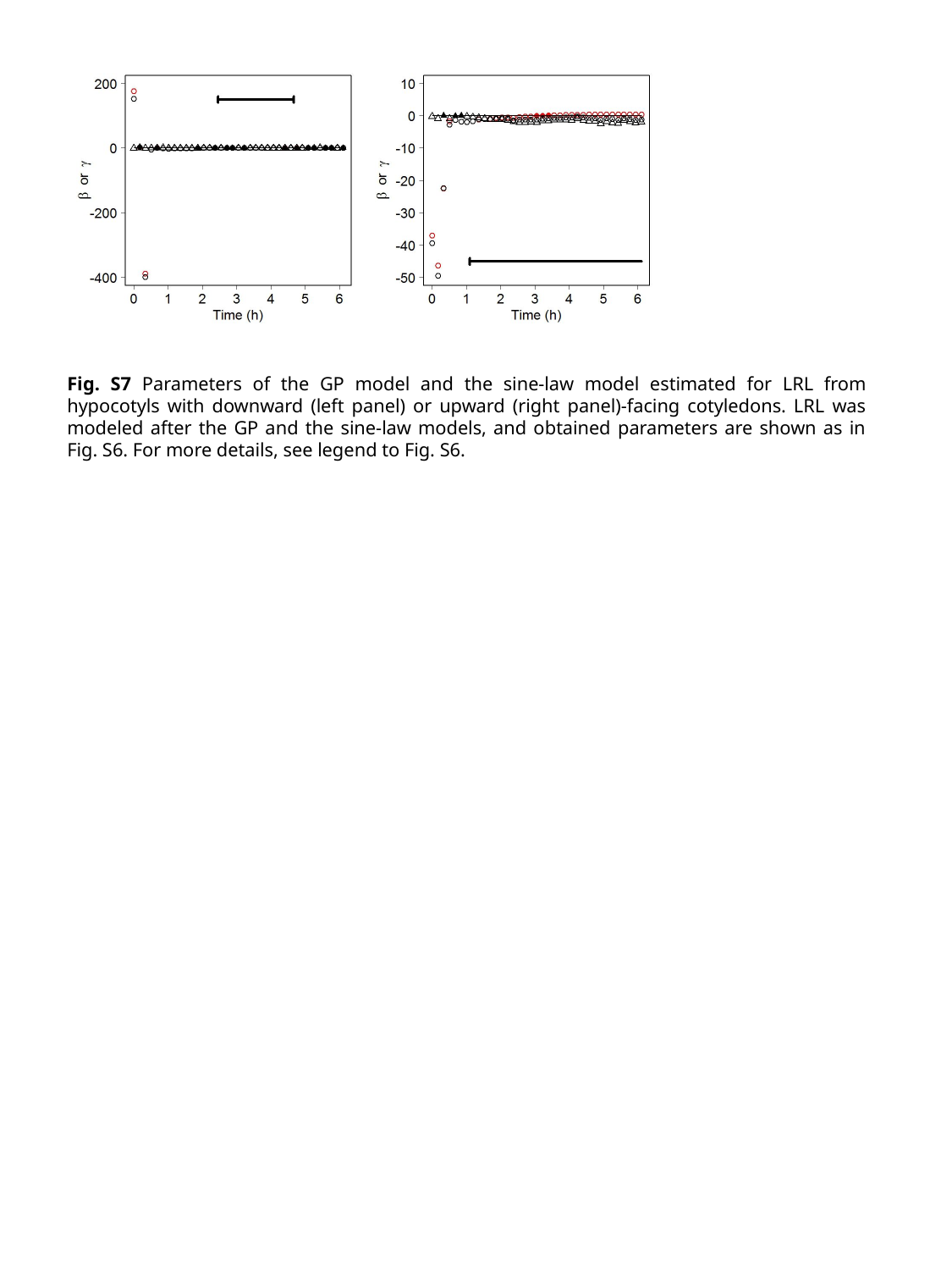

Fig. S7 Parameters of the GP model and the sine-law model estimated for LRL from hypocotyls with downward (left panel) or upward (right panel)-facing cotyledons. LRL was modeled after the GP and the sine-law models, and obtained parameters are shown as in Fig. S6. For more details, see legend to Fig. S6.

## Slide 8
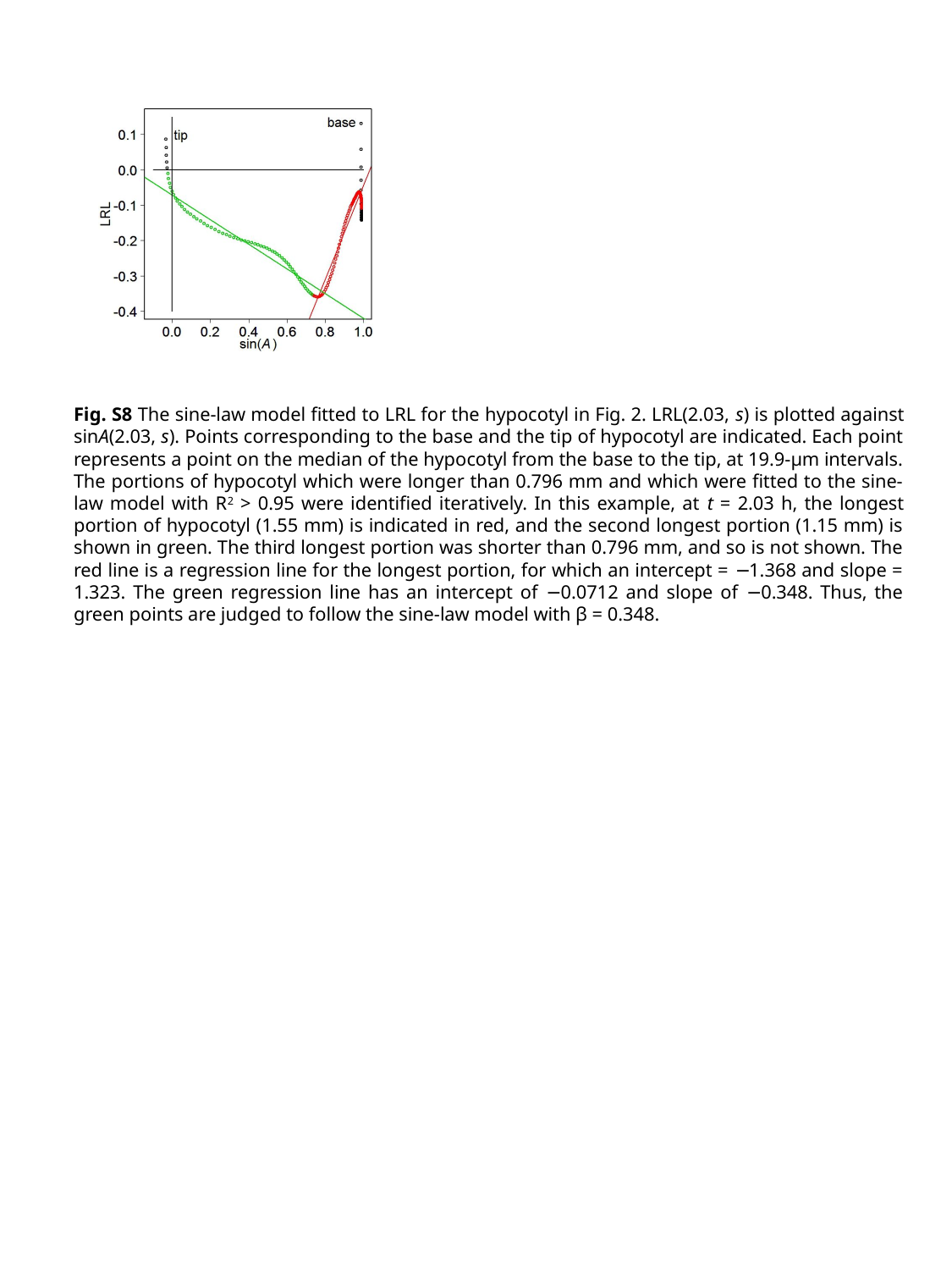

Fig. S8 The sine-law model fitted to LRL for the hypocotyl in Fig. 2. LRL(2.03, s) is plotted against sinA(2.03, s). Points corresponding to the base and the tip of hypocotyl are indicated. Each point represents a point on the median of the hypocotyl from the base to the tip, at 19.9-μm intervals. The portions of hypocotyl which were longer than 0.796 mm and which were fitted to the sine-law model with R2 > 0.95 were identified iteratively. In this example, at t = 2.03 h, the longest portion of hypocotyl (1.55 mm) is indicated in red, and the second longest portion (1.15 mm) is shown in green. The third longest portion was shorter than 0.796 mm, and so is not shown. The red line is a regression line for the longest portion, for which an intercept = −1.368 and slope = 1.323. The green regression line has an intercept of −0.0712 and slope of −0.348. Thus, the green points are judged to follow the sine-law model with β = 0.348.

## Slide 9
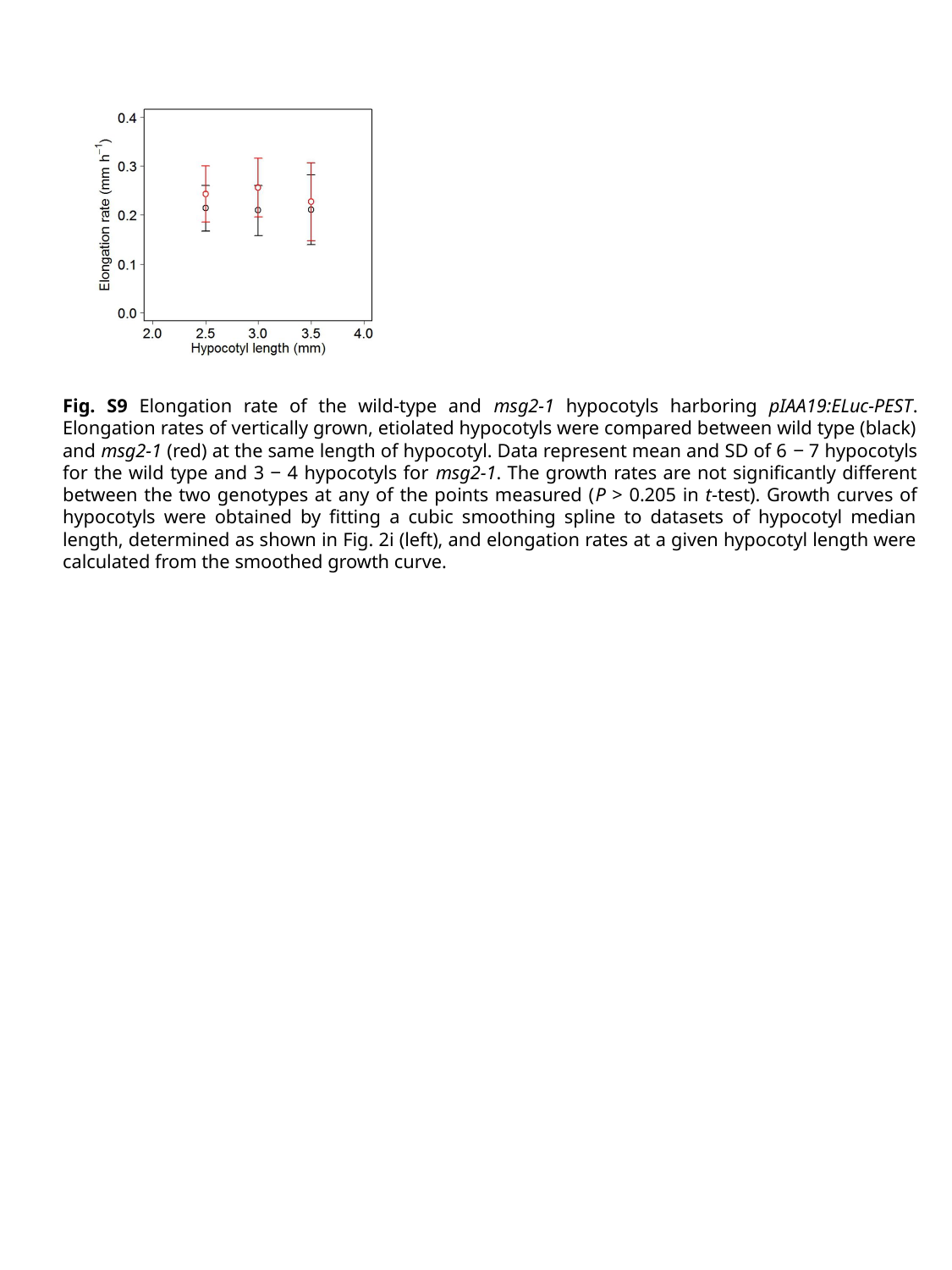

Fig. S9 Elongation rate of the wild-type and msg2-1 hypocotyls harboring pIAA19:ELuc-PEST. Elongation rates of vertically grown, etiolated hypocotyls were compared between wild type (black) and msg2-1 (red) at the same length of hypocotyl. Data represent mean and SD of 6 ‒ 7 hypocotyls for the wild type and 3 ‒ 4 hypocotyls for msg2-1. The growth rates are not significantly different between the two genotypes at any of the points measured (P > 0.205 in t-test). Growth curves of hypocotyls were obtained by fitting a cubic smoothing spline to datasets of hypocotyl median length, determined as shown in Fig. 2i (left), and elongation rates at a given hypocotyl length were calculated from the smoothed growth curve.

## Slide 10
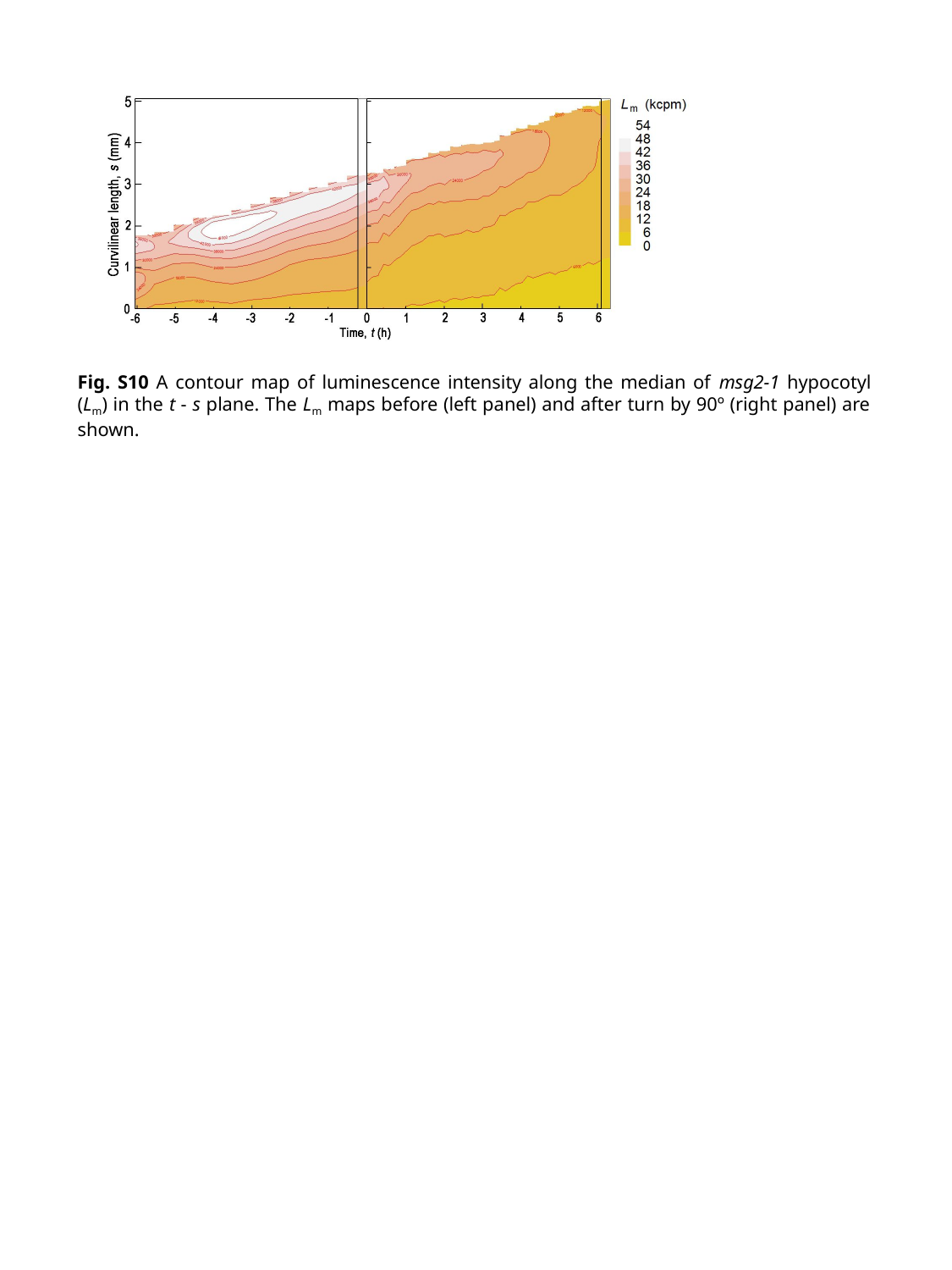

Fig. S10 A contour map of luminescence intensity along the median of msg2-1 hypocotyl (Lm) in the t - s plane. The Lm maps before (left panel) and after turn by 90º (right panel) are shown.
